# Allele frequency of *SLC*4*A*3 (PRA1), *TTC*8 (PRA2), and PRA-prcd mutations in golden retrievers in Brazil

**DOI:** 10.3389/fvets.2022.973854

**Published:** 2022-10-17

**Authors:** Anelize Souza Trecenti-Santana, Giulia Gumiero Guiraldelli, Lukas Garrido Albertino, Julia Franco Ferreira, Fabiana Michelsen Andrade, Alexandre Secorun Borges, José Paes Oliveira-Filho

**Affiliations:** ^1^School of Veterinary Medicine and Animal Science, São Paulo State University (UNESP), Botucatu, Brazil; ^2^Zootechnics Department, School of Agronomy, Federal University of Rio Grande do Sul (UFRGS), Porto Alegre, Brazil

**Keywords:** canine PRA, DNA sequence analysis, genetic disease, loss of vision, prevalence study, retinal diseases

## Abstract

Progressive retinal atrophy (PRA) is a term used in veterinary medicine to describe inherited and progressive retinal diseases characterized by progressive retinal degeneration and loss of vision. In the Golden Retriever (GR) breed, the mutations associated with PRA have an autosomal recessive inheritance pattern. This study aimed to verify the allele frequencies of PRA1, PRA2, and PRA-prcd in the GR breed in Brazil. A total of 121 GR DNA samples (*n* = 66 females and *n* = 55 males) were analyzed. All animals assessed in this study were identified as wild-type (121/121 animals; 100%) for PRA1 and PRA2 mutations; therefore, no carrier or homozygous animals were identified in this population. For the PRA-prcd mutation, 118 animals (118/121 animals; 97.52%) were wild-type. Three animals were genotyped as heterozygous for PRA-prcd (3/121 animals; 2.47%), demonstrating that this mutation is still present in some bloodlines and animals in Brazil, even with a rare prevalence. Five animals (5/121 animals, 4.2%) had a previous eye disease, which was diagnosed by a veterinarian as entropion (2 animals), keratoconjunctivitis sicca (1 animal), corneal ulcer (1 animal), and bilateral blindness (1 animal). This dog with bilateral blindness was identified as wild type homozygous for three mutations assessed in this study; therefore, blindness was not associated with the investigated mutations. In addition, the vast majority (98.3%) of Brazilian breeders assessed in this study were unaware of these mutations as a cause of blindness in the Golden Retriever. Therefore, the present study will serve to disseminate knowledge about PRA and its genetic etiologies, as well as to support future studies with other Brazilian GR populations.

## Introduction

Canine diseases have been shown to be valuable natural models for the study of many varied human conditions (1–3). Progressive retinal atrophy (PRA) is a term used in veterinary medicine to describe inherited and progressive retinal diseases characterized by progressive retinal degeneration and loss of vision (2). Several forms of PRA, genetically distinct, have been documented in more than 100 dog breeds, and while they exhibit similar clinical signs, the etiology, age of onset, and rate of progression may vary between and within breeds (2). Over 20 different PRA-causing mutations in dogs have been identified in a number of different genes (3). Some breeds may have more than one genetic form of PRA (3).

In the Golden Retriever (GR) breed, the mutations associated with PRA have an autosomal recessive character (1–3). PRA1 mutation (c.2601_2602insC) is located at the *solute carrier family* 4 [*anion exchanger*] *member* 3 (*SLC*4*A*3) gene on chromosome 37 (2). Whereas, the PRA2 mutation (c.669delA) is located at the *tetratricopeptide repeat domain* 8 (*TTC8*) gene on chromosome 8 (3). Progressive rod-cone degeneration (PRA-prcd) mutation (c.5G > A) (1), responsible for PRA in several breeds of dogs (1, 4–8), including Labrador Retriever (1, 4, 5, 7), seems to be less frequent in GR than in previous ones (2, 4, 5, 7).

Since the allele frequencies can change among poulations and canine Brazilian populations have never been studied for these mutations, this study aimed to verify the allele frequency of PRA1, PRA2, and PRA-prcd in the GR breed in Brazil.

## Materials and methods

### Ethical approval statement

This study was approved on 09 October 2017 by the Institutional Animal Care and Use Committee (205/2017–CEUA–UNESP), and samples were collected under a strict confidentiality agreement to ensure the anonymity of establishments, owners, and animals.

### Sampling and genotyping procedure

The sampling calculation was performed using OpenEpi software (version 3.0.1); given the estimated population of 41,000 GR in Brazil, the allele prevalence of mutations in GR was 7.0% (2, 3) with a 5% margin of error. A total of 121 GR DNA samples [66 females (54.5%) and 55 males (45.5%)] were used in this study. These samples were obtained from dog owners (113 samples) and a genetic database belonging to the Laboratory of Molecular Biology of the Veterinary Clinic (LBMCV) (8 samples) from São Paulo State University (UNESP), School of Veterinary Medicine and Animal Science, Botucatu, Brazil. A questionnaire was applied to characterize the animal according to pedigree information, sex, age, and clinical signs of loss of vision. The questionnaire also evaluated the knowledge of the owner about PRA mutations in the breed. Therefore, Golden Retrievers were included in the study regardless of the presence of complaints of any previous disease; however, animals that did not have the questionnaire answered by the owners were excluded from the study.

Whole blood (34 samples) and/or saliva (87 samples) samples were collected from the animals, and genomic DNA was obtained using the ReliaPrep Blood gDNA Miniprep System (Promega, Madison, WI, USA) according to the manufacturer's instructions. The samples were stored at −20°C until the PCR technique was performed.

Specific primers for amplification of the PRA1 and PRA2 mutation points were designed using online tools (Primer Express-Applied Biosystem and BLAST-Basic Local Alignment Search Tool), while the primers to amplify the PRA-prcd mutation were previously described (8) (Table 1). PCR was performed in a total of 25 μL, which contained 12.5 μL of GoTaq Green PCR Master Mix (Promega, Madison, WI, USA), 8.5 μL of nuclease-free water, 300 μM of each primer, and 2.5 μL of DNA. In addition, a non-template control reaction was performed to check for the possible presence of contamination in the PCR preparations. The amplification conditions were as follows: initial denaturation at 95°C for 5 min, followed by 40 cycles of 95°C for 15 s, 60°C (PRA1 and PRA2) or 62°C (PRA-prcd*)* for 35 s, 72°C for 30 s, and final extension at 72°C for 1 min. Amplicons were analyzed *via* 1.5% agarose gel electrophoresis, purified by magnetic beads technique, and subjected to Sanger direct sequencing. The obtained sequences and the electropherograms were analyzed using Geneious^®^ 10.0 software (Biomatters Ltd.).

**Table 1:** PCR and sequencing primer sets used in this study.

**Primer sets**	**Primer sequences (5′−3′)**	**Product (bp^a^)**	**Melting**
PRA1	GTAGTTGGATGTCGTGGAATACT CCGGCCTGATTAAACCTCTT	473	60 °C
PRA2	CTCTGGTCTGGAACCGATTATTT GGATCTGAAGGTGCAGATT	493	60 °C
PRA-prcd^b^	CCAGTGGCAGCAGGAACC CCGACCTGCTGCCCACGACTG	512	62 °C

a Base pairs,

b Primers previously describe (8).

The allele frequency and standard error for each mutation found were estimated using the following equations: $\text{allele frequency}=\frac{\text{total number of mutant alleles }/\text{total number of animals}}{2}$ and $\text{standard error }=\sqrt{\left(2\right)}\frac{\text{allele frequency}{\;}^{*}\left(1-\text{ allele frequency}\right)}{2{\;}^{*}\text{sample size}}$. The chi-square test was used to test whether alleles of the three mutations were in Hardy-Weinberg equilibrium (HWE) within the GR population assessed, which was not consistent with HWE if *P* < 0.05.

## Results

Considering the 95% confidence interval, the minimum sample size needed to verify the allele frequency of PRA1, PRA2, and PRA-prcd in the GR breed in Brazil was 100 GR. According to the applied questionnaire, 10.7% (13/121 animals) were <1 year old, 6.6% (8/121 animals) were 1 year old, 15.7% (19/121 animals) were 2 years old, 19% (23/121 animals) were 3 years old, 13.2% (16/121 animals) were 4 years old, and 34.7% (42/121 animals) were 5 years old or more.

Five animals (5/121 animals, 4.2%) had a previous eye disease, which was diagnosed by a veterinarian as entropion (2 animals), keratoconjunctivitis sicca (1 animal), corneal ulcer (1 animal), and bilateral blindness (1 animal). The majority of the owners (119/121 owners, 98.3%) did not know if any other animal from the litter had any eye disease, and only 5.8% (7/121 owners) knew about PRA in the GR breed.

**Figure 1:**
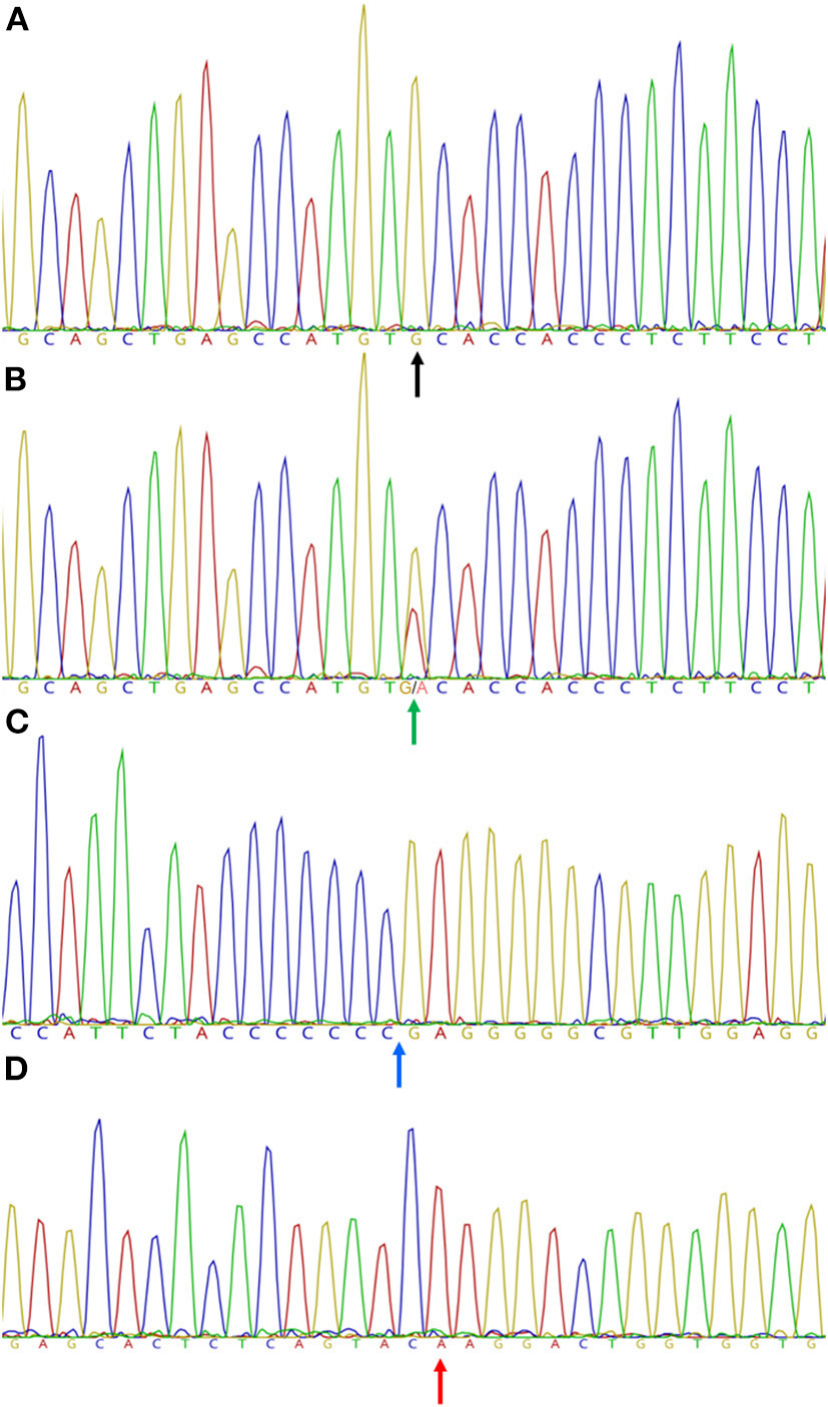
Partial chromatogram showing capillary sequencing results for homozygous wild-type **(A)** and heterozygous **(B)** mutant alleles of the (c.5G > A, PRA-prcd) mutation in the *PRCD* gene, homozygous wild-type **(C)** allele of the (c.2601_2602insC; PRA1) mutation in the *SLC4A3* gene, and homozygous wild-type **(D)** allele of the (c.669delA; PRA2) mutation in the *TTC8* gene in Golden Retrievers dogs. **(A)** wild-type allele (guanine) (black arrow); **(B)** double peak (guanine/adenine) (green arrow); **(C)** 8th cytosine insertion point (blue arrow); **(D)** point of an adenine deletion (red arrow). Images were obtained using Geneious^®^ 10.0 software (Biomatters Ltd., Auckland, New Zealand).

According to the obtained sequences and the electropherograms, all animals assessed in this study (121 animals) were wild-type for PRA1 and PRA2 mutations. For the PRA-prcd mutation, 118 animals (118/121 animals; 97.5%) were wild-type, and 3 animals (3/121 animals; 2.5%) were heterozygous (carrier) for the mutation (Figure 1). Therefore, the PRA-prcd allele frequency was 0.012 ± 0.007, while for the other two mutations it was zero. The alleles distribution of the PRA1 and PRA2 SNP were not consistent with HWE, while the PRA-prcd allele frequency was in equilibrium in the GR group (*P* = 0.899).

In addition, the three GR carriers of the PRA-prcd mutation were from different cities and distinguished owners and were not related to each other, and the only dog with bilateral blindness was identified as wild type homozygous for all three mutations assessed in this study. Blindness was confirmed during the DNA sampling, but the dog's owner preferred not to invest in the diagnosis of possible causes after receiving the result of these genotyping.

## Discussion

The present study is the first report of the prevalence of PRA1, PRA2, and PRA-prcd mutations in the Brazilian GR breed. Although these mutations have not been described in Brazil, studies in other countries contributed to defining the importance of these mutations in the GR breed (2–4) and as valuable natural models for the study of many varied human conditions (1–3).

Reports of PRA-prcd mutations in the GR breed are rare (2, 3, 9–11). According to the Golden Retriever Club of America (9), the prevalence of this mutation in the breed was 0.06% from 1990 to 1999 and 0.05% from 2000 to 2005. After 2007, with the knowledge of the mutations that cause PRA and the introduction of commercial genetic tests, no PRA-prcd causative mutation was found in GR dogs (9). In addition, according to the Golden Retriever Club (10), this mutation is virtually eliminated from the GR population in the UK, but no data are presented.

The PRA1 mutant allele frequency was 0.613 ± 0.039 in the group of PRA-affected GR dogs assessed by Downs et al. (2). On the other hand, the frequency of the PRA1 mutant allele was 0.056 ± 0.008 in the GR group known to be free of PRA, which is nine times lower than that found in PRA-affected GR (0.056 vs. 0.613) (2). In another study, Downs et al. (3) described the PRA2 mutation as the cause of PRA in GR dogs, and by genotyping 2,500 GR dogs for this mutation, the authors found the PRA2 mutant allele frequency to be 0.040 ± 0.003. In addition, when these authors genotyped GR PRA affected that were not caused by PRA-prcd or PRA1, the PRA2 mutant allele frequency increased 19 times (0.776 ± 0.055) (3).

Even using a larger number of samples than the minimum sample size calculated (121 vs. 100), unlike these studies, the PRA 1 and PRA2 mutations were not found in the Brazilian GR evaluated in the present study. PRA1 allele frequencies were 0.06 in Sweden (109 GR assessed), 0.04 in the UK (108 GR assessed) and 0.02 in France (89 GR assessed), but the variant has not been found in 148 GR from the USA (2). The PRA2 allele frequencies were 0.045 in Denmark (132 GR assessed), 0.04 in Sweden (736 GR assessed), 0.03 in France (87 GR assessed), 0.01% in the UK (88 GR assessed) and 0.01% in the USA (179 GR assessed) (3). These authors believe that the presence of the PRA2 variant in GR dogs in these countries suggests that the PRA2 variant may have arisen prior to the geographic dispersion of the breed (3). According to the CBRGR (Brazilian Council for Golden Retriever Breed) (12), the Brazilian GR genetic consists mainly of blood lineages from the USA. In this sense, we hypothesized that the lack of detection of PRA1 and PRA2 mutant alleles in Brazilian GR that did not affect PRA may be due to our samples being composed mainly of dogs from USA blood lineages, where PRA1 has not been found (2) and the PRA2 allele frequency was considered low (3).

Downs and colleagues (2) tested 80 GRs with PRA for the PRA-prcd mutation and found only one heterozygous animal (allele frequency 0.006 ± 0.006) among them. In contrast to these findings, in the present study, only animals without signs of PRA were evaluated, and although the PRA-prcd allele frequency (0.012 ± 0.007) observed was low, it was higher than those observed in these studies (2, 5).

As stated before, the PRA1 (with the exception of the USA) and PRA2 mutations were found in the majority of GR PRA affected (2, 3), while PRA-prcd mutation seems to be rare in this breed, being found only in heterozygosis (2). On the other hand, the genetic explanation for ~9% of PRA cases remains to be identified (3). In this sense, although PRA1 and PRA2 mutations were not found in the present study, it is difficult to say precisely that these, especially PRA2, are not present in Brazil, since GRs suffering from PRA were not evaluated in the present study.

Additionally, our greatest concern in preventing the disease in Brazil is knowing that the vast majority (98.3%) of the owners of the GR assessed in the present study were unaware of the disease and its possible causes. Furthermore, according to the literature consulted, we are not aware of reports of GR diagnosed with PRA in Brazil, and this may be another obstacle in the prevention of the disease in Brazil, since PRA may be underdiagnosed in the small animal clinical routine. Therefore, the present study will serve to disseminate knowledge about PRA and its genetic etiologies, as well as to support future studies with other Brazilian GR populations.

## Data availability statement

The original contributions presented in the study are included in the article/supplementary material, further inquiries can be directed to the corresponding author.

## Ethics statement

The animal study was reviewed and approved by Institutional Animal Care and Use Committee from School of Veterinary Medicine and Animal Science. Written informed consent was obtained from the owners for the participation of their animals in this study.

## Author contributions

AT-S, JO-F, and FA contributed to the conception and design of the study. AT-S, GG, LA, JF, and FA contributed to the sample collection. AT-S, GG, LA, and JF contributed to sample processing. AT-S, LA, AB, and JO-F contributed to writing the first drafts of the manuscript. JO-F contributed to directing the project. All authors contributed to manuscript revision and read and approved the submitted version.

## Funding

This study was funded by the São Paulo Research Foundation (FAPESP, Brazil), Grant Number 20/15284-8.

## Conflict of interest

The authors declare that the research was conducted in the absence of any commercial or financial relationships that could be construed as a potential conflict of interest.

## Publisher's note

All claims expressed in this article are solely those of the authors and do not necessarily represent those of their affiliated organizations, or those of the publisher, the editors and the reviewers. Any product that may be evaluated in this article, or claim that may be made by its manufacturer, is not guaranteed or endorsed by the publisher.
